# Auto-generated materials database of Curie and Néel temperatures via semi-supervised relationship extraction

**DOI:** 10.1038/sdata.2018.111

**Published:** 2018-06-19

**Authors:** Callum J. Court, Jacqueline M. Cole

**Affiliations:** 1 Cavendish Laboratory, Department of Physics, University of Cambridge, J.J. Thomson Avenue, Cambridge CB3 0HE, UK; 2 ISIS Neutron and Muon Source, STFC Rutherford Appleton Laboratory, Harwell Science and Innovation Campus, Didcot, Oxfordshire OX11 0QX, UK; 3 Argonne National Laboratory, 9700 South Cass Avenue, Argonne, IL 60439, USA; 4 Department of Chemical Engineering and Biotechnology, University of Cambridge, West Cambridge Site, Philippa Fawcett Drive, Cambridge CB3 0FS, UK

**Keywords:** Magnetic properties and materials, Magnetic properties and materials, Cheminformatics, Magnetic materials, Solid-state chemistry

## Abstract

Large auto-generated databases of magnetic materials properties have the potential for great utility in materials science research. This article presents an auto-generated database of 39,822 records containing chemical compounds and their associated Curie and Néel magnetic phase transition temperatures. The database was produced using natural language processing and semi-supervised quaternary relationship extraction, applied to a corpus of 68,078 chemistry and physics articles. Evaluation of the database shows an estimated overall precision of 73%. Therein, records processed with the text-mining toolkit, ChemDataExtractor, were assisted by a modified Snowball algorithm, whose original binary relationship extraction capabilities were extended to quaternary relationship extraction. Consequently, its machine learning component can now train with ≤ 500 seeds, rather than the 4,000 originally used. Data processed with the modified Snowball algorithm affords 82% precision. Database records are available in MongoDB, CSV and JSON formats which can easily be read using Python, R, Java and MatLab. This makes the database easy to query for tackling big-data materials science initiatives and provides a basis for magnetic materials discovery.

## Background & Summary

**Figure 1:**
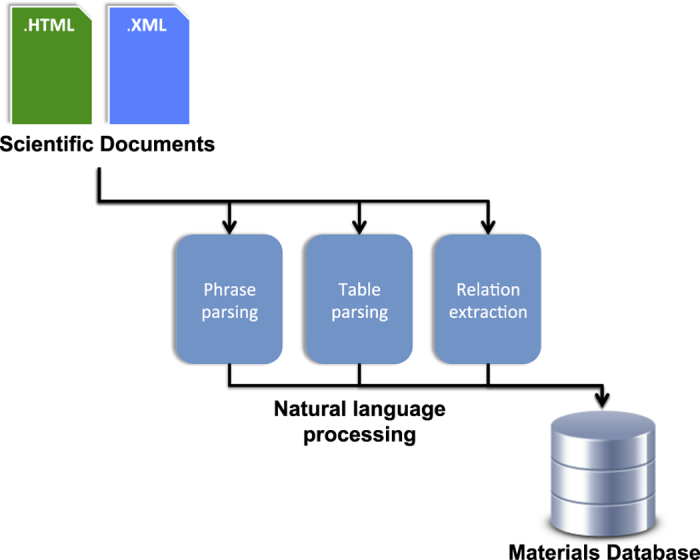
Overview of the operational workflow of our automated text-mining system. Starting with a corpus of scientific articles in a variety of file formats, the system uses advanced natural language processing techniques to automatically extract magnetic properties with the chemical formula or identifier of the associated material. These paired entities are then automatically compiled into a highly structured materials database.

Within the sub-fields of materials science, journal articles, patents and theses form a vast and continually growing corpus of unstructured information. The large volume of previously generated data, and the growing rate of publication, makes it impossible for researchers to make full use of the available information^1^. Compiling these data into structured materials databases would enable data-driven research to be performed with the goal of materials discovery, but doing so manually is highly time consuming and prone to human generated error^2^. Advances in Natural Language Processing (NLP) and Machine Learning (ML) techniques have made possible the automated data mining from text and tables across vast numbers of documents. Such data mining capabilities can be used for auto-generating highly structured materials property databases (Fig. 1).

There have been a variety of big-data projects for materials discovery, spearheaded by the Materials Genome Initiative^3^. Such projects include the Harvard Clean Energy Project, which employs neural network style architectures to perform research in the field of organic photovoltaics^4^, generating 2.6 million data points for candidate compounds^5^. Similarly, the Materials Project^6^ uses *ab-initio* computational techniques to generate prospective battery materials properties. As of November 2017, the Materials Project database contains 69,640 inorganic compounds and 530,243 nanoporous materials^7^.

In any field of materials discovery, the ability to auto-generate materials property databases in a domain independent manner would be highly beneficial. It would enable researchers to quickly and easily produce materials databases that are tailored to their custom research needs and without the need for the large domain-specific datasets. The ChemDataExtractor^8^ toolkit provides a means of creating domain-independent materials property databases by extracting data from the scientific literature. Using a hybrid pipeline of NLP and ML techniques, the system is able to extract a wide variety of chemical structures and properties with high precision and recall.

Previous work on automated information retrieval of materials science data via text mining has primarily targeted the organic chemistry literature for chemical and biomedical applications^9^. In stark contrast, little work has been done to automatically collate information about inorganic compounds and their properties, save for one recent study that has extracted synthetic parameters for inorganic oxide materials^10^. The application of automated text-mining methods to create databases of physical properties of inorganic materials has not hitherto been reported. This is despite the fact that the application of big data and ML techniques, to fields such as magnetism and superconductivity, could have great implications for research into data storage devices, quantum information processing and medicine. At the current time, only manually curated databases exist for magnetic materials, which are very modest in dataset size^11,12^ or are not open source and have been designed for single-entry look up^13^ making it difficult to apply data science to them.

To this end, this work presents a fully auto-generated open-source database of 39,822 Curie and Néel phase transition temperature records. These properties describe the temperatures at which magnetic compounds undergo ferromagnetic and antiferromagnetic phase transitions, respectively. These properties were chosen to form the basis for further work on magnetic materials discovery as they are commonly reported properties of newly synthesised compounds in both chemistry and physics journal articles.

The data records were automatically extracted from a corpus of 68,078 journal articles using a newly adapted version of the ChemDataExtractor toolkit. The adapted system incorporates a semi-supervised, probabilistic and quaternary relationship extraction stage based largely on the Snowball relationship extraction algorithm^14^. ChemDataExtractor (version 1.3) has shown >90% precision in data extraction of organic materials properties. However, the built-in phrase parsing rules within ChemDataExtractor 1.3 were found to yield a precision of only 66% when applied to extracting magnetic phase transition temperatures from typical inorganic chemistry and physics journal articles. To remedy this, a modified Snowball algorithm was designed to extract relationships probabilistically from text that varies only slightly from the pre-trained rules. Introducing this algorithm as a new stage within the NLP pipeline of ChemDataExtractor is shown to improve the precision of data extraction on previously unseen text. The resulting database records are available in MongoDB, CSV and JSON formats, which can easily be read for the purpose of magnetic property prediction and discovery.

## Methods

### Acquiring a corpus of data sources

The corpus of 68,078 articles was produced using web-scraping tools incorporated within the scrape package of ChemDataExtractor 1.3. These scraping tools allow the user to submit a query to the search pages of the Royal Society of Chemistry and Springer publishers to retrieve all the relevant journal articles. In this work, the scrape package was further extended to include similar scrapers for the SpringerLink platform and the Science Direct webpages by Elsevier, designed to work alongside their Text and Data Mining Application Programming Interface (API). The corpus was obtained by submitting queries for “Néel+temperature'' and “Curie+temperature'' to all accessible journals from these publishers and retrieving the full-text articles in either HTML or XML format.

### Data extraction

**Figure 2:**
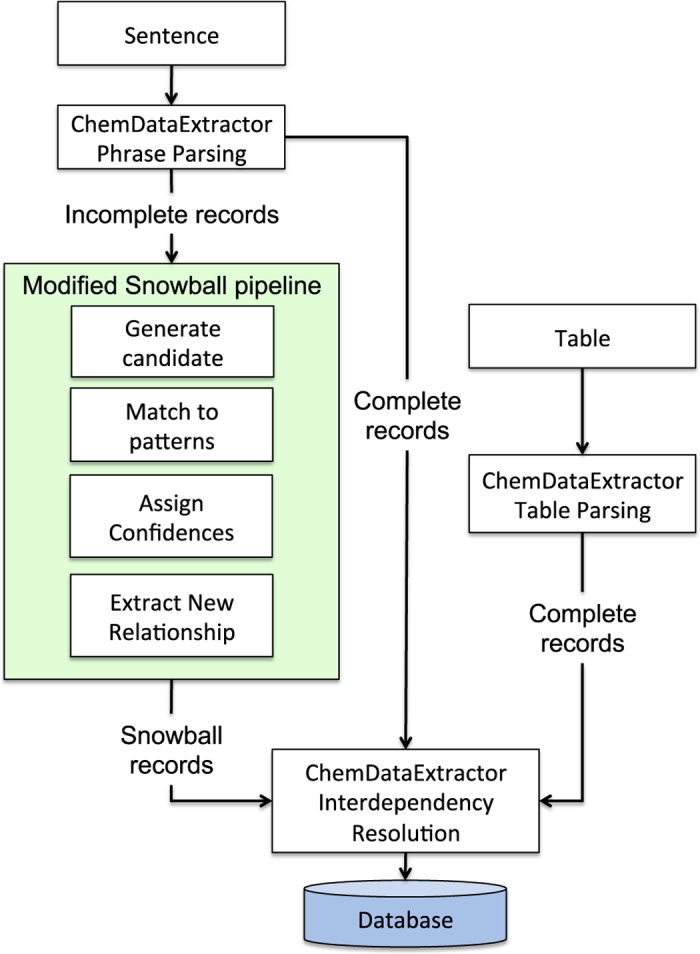
The combined ChemDataExtractor and modified Snowball pipeline. Article sentences are parsed with the built-in ChemDataExtractor phrase parsers. Any incomplete records that contain properties but no associated compounds are passed to the modified Snowball algorithm pipeline as a candidate for relationship extraction. The candidate sentence is split into its elements and vectorised to form a candidate phrase object. This is then compared to the pre-trained extraction patterns. A similarity measure between the candidate phrase object and these extraction patterns is used to assign a confidence score using Equation 5. If the confidence score is sufficiently high, the relationship within the candidate phrase object is accepted. All complete records are then passed through the ChemDataExtractor interdependency resolution stage and added to the database.

The extraction of chemical records from each document was achieved via a pipeline of ML and NLP techniques. Fig. 2 provides a schematic overview of the operational workflow.

The articles were first processed using the ML and NLP pipeline of ChemDataExtractor as described in ref. 8. These stages convert XML and HTML documents into standardised formats and use rule-based phrase and table parsing in combination with probabilistic Named Entity Recognition (NER) methods to extract chemical relationships. In this work, we added two new parsers to ChemDataExtractor for the extraction of Curie and Néel phase transition temperatures. Evaluation of the current release of ChemDataExtractor, which uses only rule-based phrase parsing for data extraction, yielded an F-score of 66%, far lower than its performance on organic chemistry text and tables. This low F-score of data extraction on inorganic chemistry and condensed matter physics literature was determined to be caused by a number of common themes that make this type of text different to the organic chemistry literature.

Firstly, it was found that although the Chemical Named Entity Recognition (CNER) system within ChemDataExtractor 1.3 is good at extracting chemical compound names and labels from typical organic chemistry text, it struggles when presented with compound labels that are more common in the inorganic chemistry or physics literature. For example, it fails on chemical formulae including doping concentration labels, such as *“In Ba*_*x*_*Mn*_*1−x*_*O*3*, TN=200 K for x=0.9''*. In cases such as this, where the doping concentration is given separately, either the compound name is missed entirely or the Néel temperature value is not correctly associated to it. In this work, a set of new Chemical Entity Mention (CEM) parsing rules have been added to extract the correct compound names and labels from these cases.

It was also noted that chemical properties are often presented for a series of compounds within a single article. In these cases, it is common to label each sub-compound with a numeric label (often within parentheses) rather than to refer to a compound by its chemical formula. For example, in the introduction of a journal article, a compound may be given in the form *“...[Ni3(cmpa)2(N3)4(H2O)2]n5nH2O (1)..''*, then later in the document, “*(1) shows antiferromagnetic ordering at TN = 6.5K*.''. The default ChemDataExtractor rules for detecting compound labels were found to be too restrictive to handle these cases. The label detection rules have now been adapted so that a single numerical label can be used to define a chemical relationship.

Other phrase parsing rules were updated to incorporate typical CEM suffix words associated with magnetic compounds, such as *ferroelectrics*, *ferrites*, *orthoferrites*, *nanotubes*, *nanocrystals*, *thin-films*, *quantum-dots* and *ceramics*. These were designed in such a way that the individual words do not constitute a compound name, but will be identified alongside a compound to produce more specific records. Similarly, CEM prefix word parsers, such as *bulk*, *amorphous*, *crystalline*, *pure* and *doped* were added.

Another common theme in the magnetic property literature is to use an informal chemical symbol to refer to a class of elements, such as *Ln*, to identify lanthanides or *RE* to indicate rare-earth metals. Therefore, more rules were added to identify these informal chemical labels and associate them to properties accordingly. All these changes ensure that records output to a database are more precise and give the user a clearer picture of the compound in question.

Despite these changes to the phrase-parsing rules within ChemDataExtractor, the rule-based approach to phrase parsing has a few limitations. Firstly, whenever a user wishes to update ChemDataExtractor to extract new properties, a whole new set of rules must be programmed. This requires a large amount of trial and error to generate successful rules. Furthermore, the information extraction is guaranteed to fail on sentences that contain only minor variations from the rules. Therefore the rules must be both specific enough to capture minor language variations and general enough to increase the recall of the system. This problem highlights one of the many issues with completely deterministic information retrieval from text.

The performance of the purely rule-based phrase parsing approach is ultimately limited by the large variation in the textual language used to specify chemical properties within journal articles. As such, the rule-based parsers implemented in ChemDataExtractor 1.3 yield lower precision and recall of data extraction on inorganic materials. This motivates the need for a relationship extraction system that is able to identify magnetic property relations from text in a probabilistic way. Such a system would be able to retrieve relationships from text that varies from pre-programmed rules and provide a confidence score to each record, giving the user a measure of record validity that was not previously available with ChemDataExtractor.

### Training a semi-supervised relationship extraction algorithm

In this work, the operational workflow of ChemDataExtractor has been extended to incorporate a semi-supervised and probabilistic ML algorithm for quaternary relationship extraction. Since ChemDataExtractor contains trained CNER tools, it is well suited to incorporating a semi-supervised approach to relationship extraction such as the Snowball algorithm^14^. The original Snowball algorithm operates as follows: it uses approximately 4,000 seed examples of Organisation-Location pairs to perform binary relationship extraction. The process starts with a set of seed examples of a known positive relationship, each assigned a full confidence score of 1.0. The algorithm is then trained by parsing a corpus of documents to find sentences therein which contain the seed examples. These sentences are clustered based on textual similarity and used to learn typical patterns that specify the relationship. New relationships can be identified by comparing previously unseen sentences to these learnt patterns and checking for a minimum level of similarity. This level of similarity also helps to determine the confidence score of the new relationship.

**Figure 3:**
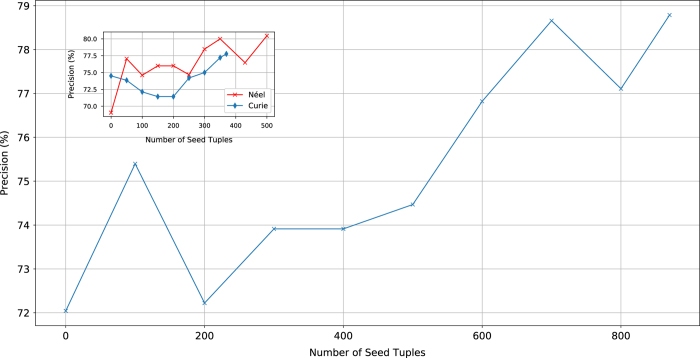
Learning curve of the modified Snowball algorithm pipeline as a function of combined Néel and Curie temperature seed tuples. The number of seed tuples used to train the modified Snowball algorithm stage were varied from 0 to 500 for the Néel temperature clusters and from 0 to 370 for the Curie temperature clusters. At each stage, seed tuples from both sets were chosen at random in increments of 50. For each number of seed tuples, the full extraction pipeline was applied to a randomly selected corpus of 50 papers to produce approximately 100 records. The resulting records were compared to the source articles to determine the record precision, using Equation 6. The result shows a roughly linear increase in precision with the number of seed tuples, giving a 9% relative improvement in precision over completely deterministic phrase parsing. Inset: The precision curves for Néel and Curie temperature records evaluated separately.

The original Snowball algorithm demonstrated high precision, over 90% for simple binary relationship extraction^14^. However, for extracting chemical relationships, the algorithm must be generalised to extract quaternary relationships consisting of entities such as: a property specifier (e.g Néel temperature, *T*_*N*_, Curie temperature, *T*_*C*_ etc.), a CEM or label, the property value and the property unit. Accordingly, a modified Snowball algorithm that employs quaternary relationship extraction was constructed and incorporated into the operational workflow of ChemDataExtractor as demonstrated in Fig. 2. Figure 3 shows the methodology of the modified Snowball algorithm alongside an illustrative example in the right-hand column.

The first stage of the process is to curate a set of training seed tuples that comprise quaternary relations, e.g. chemical name, property specifier, property value and property unit. The seeds in this case study were found by manually analysing a training corpus of 400 articles from chemistry and physics journals. Care was taken to ensure no duplicate seed tuples were generated to avoid any biases in the confidence scoring stages. However, if sentences were found with matching compound names but different property values or units, these were taken to constitute different tuples. Similarly, abbreviations or different names for the same compounds were also taken to be different tuples. For example, this means that *(Néel, Bismuth Ferrite, 640, K)*, *(Néel, BFO, 370, C)* and *(Néel, BiFeO3, 640, K)* are all considered to be different tuples.

Training the algorithm begins by scanning all of the training corpus to find the sentences that contain any of the seed tuples. Using the example that illustrates the operational workflow of the algorithm (Fig. 3), this stage returns sentences such as, *“It was found that BiFeO3 and LaFeO3 have Néel temperatures of 640 and 750 K, respectively"*, which contains two seed tuples, *(Néel, BiFeO3, 640, K)* and *(Néel, LaFeO3, 750, K)*.

Next, the detected sentences are clustered into a two-stage hierarchy. To account for sentences that contain multiple seed relations, the sentences are first clustered based on the order and number of named entities. This assigns each sentence a label describing the entity ordering. In the example given in Fig. 3, this stage identifies the entity order as being two CEMs (BiFeO3, LaFeO3), followed by one property specifier (Néel), two values (640, 750) and one unit (K); yielding a total of 6 entities. All sentences with the same order and number of entities are clustered together.

Each sentence that generated these entity ordering clusters is tokenised by splitting the sentence on whitespace, creating a list of tokens corresponding to individual words, punctuation, numbers or symbols. The list of tokens is then split into three elements: the prefix, middles and suffix. The prefix is the series of tokens preceding the first entity, the middles are the tokens between each entity, and the suffix is the series of tokens after the last entity. The result is a phrase object containing *m* entities, *e*_*i*_, and *m*+1 of these phrase elements, in the following form:

(1)Phraseobject={prefix,e1,middle1,e2,...,middlem−1,em,suffix}

Continuing with the example in Fig. 3, the phrase object created for the given sentence takes the general form:

(2){“that”,e1,“and”,e2,“have”,e3,“of”,e4,“and”,e5,e6,“respectively”}

where *e*_1_=*BiFeO*3 and *e*_2_=*LaFeO*3 are CEM entities, *e*_3_=*Néel temperatures* is a specifier entity, *e*_4_=640 and *e*_5_=750 are value entities and *e*_6_=*K* is a unit entity. The number of tokens for the prefix and suffix can be set by the user to be as long or short as possible. In this system, they were fixed to a maximum of 1 token.

At this stage, these phrase objects have been assigned to clusters based on the ordering and number of entities. Next, sub-clusters are generated using a single pass classification algorithm^15^ which clusters phrases based on their level of textual similarity.

Before clustering can take place, the elements of each phrase object (prefix, middles and suffix) must be vectorised into a normalised vector of token weights. This is achieved using a Term Document Frequency (TDF) model^16^ that assigns a weight to a token equal to the number of times that token appears within a specific element of all phrase objects in the ordering cluster, normalised by the total number of tokens. This means that a token appearing frequently in the phrase objects has a greater weight than tokens appearing infrequently.

Calculating the similarity between phrases works as follows. For two phrase objects *p* and *q*, each containing *m*+1 normalised vector elements in the form of Equation 1, the similarity is:

(3)sim(p,q)=∑im+1vi(pi⋅qi)

where (*p*_i_ *q*_*i*_) is a simple dot product between element vectors and *v*_*i*_ is an importance weight that determines the contribution of each element to the overall similarity, e.g. the prefixes and suffixes can contribute less to the similarity score than the middles. This is a parameter of the system that can be varied by the user. The output is a similarity score between 0 and 1. Following the single pass clustering algorithm detailed in ref. 15, phrase objects are then assigned to sub-clusters based on this similarity measure. The first phrase is assigned to its own sub-cluster, then each subsequent phrase is assigned to a cluster if the similarity between it and the sub-cluster extraction pattern is above the minimum similarity threshold, *τ*_*sim*_.

The sub-cluster extraction pattern represents the phrase object most similar to all others. When a new phrase object is added to a cluster, the extraction pattern is updated to reflect the new information. The essence of this extraction pattern generation stage comprises determining the most common prefix, middle and suffix across all phrase objects in the sub-cluster and subsequently combining these elements to form a new phrase object.

As with the seed tuples, the extraction patterns must be assigned confidence scores. The scores are chosen to reflect the frequentist likelihood that an extraction pattern produces correct relationships. The scoring method used is the same as the approach used in the original Snowball algorithm. The extraction pattern, *P*, is applied to all sentences in the training corpus that were found to contain seed tuples. The confidence, *C*(*P*), is then calculated using:

(4)C(P)=NumberofpositivematchestoseedtuplesentencesTotalnumberofmatchestoseedtuplesentences

where a positive match occurs if the extraction pattern correctly retrieves the seed tuple(s) from the original sentence.

In this work, this process was applied to learn extraction patterns for Curie and Néel phase transition temperatures. A training corpus of 400 journal articles were examined to extract 500 Néel and 370 Curie seed tuples, respectively. Each seed tuple was assigned a confidence score 1.0. The training process then generated a total of 412 and 194 extraction patterns, respectively; Table 1 demonstrates a few examples of the learnt extraction patterns.

The precision (Equation 6) of the extracted relationships is largely dependent on the number of seed tuples used to train the modified Snowball algorithm. Using more seed tuples increases the number of phrases generated by the algorithm and hence increases the coverage of the resulting extraction patterns. Figure 3 shows the learning curve of the system (in terms of precision) as a function of the number of seed tuples used to train the modified Snowball algorithm. The number of seeds were varied in increments of 100, from 0 (corresponding to data extraction using purely phrase parsing) to 870 (the maximum combined number of manually derived Curie and Néel seed tuples). For each number of seed tuples, the modified Snowball algorithm pipeline was applied to a set of 50 randomly chosen articles from the corpus. The precision of the resulting records was then calculated by comparing the records to the source articles. The learning curves show an approximately linear increase in precision with the number of seed tuples; from 72.0% using only phrase parsing, to 78.8% when using the fully trained modified Snowball algorithm. This represents a 9.4% relative improvement in precision. The continuing linear increase in precision indicates that the system could be further improved by curating more seed tuples. However, the time taken to manually curate large numbers of seed tuples would naturally negate the aim of automated materials property database generation.

### Finding new relationships

**Figure 4:**
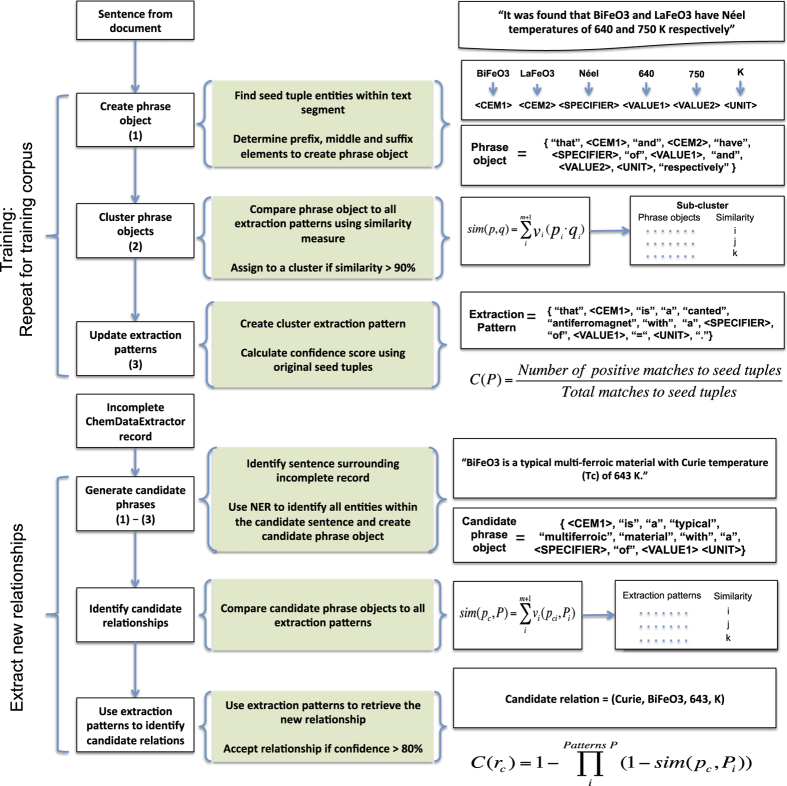
Example of the modified Snowball algorithm pipeline. To train the algorithm, a training corpus is parsed to find all occurrences of the manually generated seed tuples. These tuples comprise quaternary relations that have been extracted from the sentence. They are then clustered based on the similarity measure given in Equation 3. Each phrase cluster generates an extraction pattern that represents the phrase most similar to all others within the cluster. To learn new relationships from previously unseen text, candidate sentences are compared to the extraction patterns and scored based on their level of similarity. The resulting relationships are accepted provided their confidence is above a pre-determined threshold.

After training, the system can be used to find new relationships that have been mis-identified by ChemDataExtractor. The overarching process of this was outlined in Fig. 2. This showed that, following the phrase parsing stage of ChemDataExtractor, any incomplete records are passed onto the modified Snowball pipeline stage. In the case study, such incomplete records are those that have identified a Curie or Néel temperature but have not found an associated compound name or label. Likely reasons for an incomplete record are: the original sentence did not match to a parsing rule, or, the sentence does not contain a compound name or label. Therefore, the modified Snowball pipeline, detailed in Fig. 4 first checks the sentence, from which the incomplete record was derived, to see if it contains all the required entities to form a Curie or Néel relationship (compound names, values, units and property specifiers). Any sentence matching this criteria is taken to be a candidate sentence for containing a Curie or Néel relationship.

The candidate sentence is converted to a vectorised candidate phrase object, which is compared to all extraction patterns using the same similarity measure as given in Equation 3. An example candidate phrase object is shown in Fig. 4. If the candidate phrase object matches to one or multiple extraction patterns with a similarity score above a pre-set threshold, *τ*_*c*_, then these extraction patterns are used to identify candidate relations from the candidate phrase.

The confidence score of a candidate relation, *r*_*c*_, derived from a candidate phrase object, *p*_*c*_, is calculated using the confidence scores of these extraction patterns using Equation 5.

(5)C(rc)=1−∏P[1−C(Pi).sim(pc,Pi)]

This confidence scoring scheme ensures that a candidate phrase object matching to many extraction patterns with high similarity, has higher confidence than one which matches to few patterns with low similarity. Finally, if the confidence score is above a pre-defined threshold score of 80% the candidate relation is accepted.

This concludes a single pass of the algorithm as shown in Fig. 4. However, it is important to mention that a positive feedback loop is in effect. To that end, the candidate phrase from which the new relation was derived is added to the sub-clusters, and the extraction patterns are updated to reflect this new information. The updating process ensures that the system continually adapts the extraction patterns as it is presented with previously unseen sentences and gradually improve recall and precision of data extraction over time.

As shown in Fig. 2, all records that are output from the phrase parsing stage, table parsing stage and modified Snowball pipeline are passed through the ChemDataExtractor interdependency resolution stage, as described in ref. 8. Since the modified Snowball algorithm can only resolve sentence-level relationships, some incomplete records cannot be completed by this stage of the pipeline. For example, the appropriate chemical identifier may occur in the preceding paragraph or section. Therefore, any records that are still incomplete after the modified Snowball pipeline are processed with the ChemDataExtractor interdependency resolution stages to find any global contextual information from the preceding paragraphs.

### Post-processing

Once the records have been extracted, the data are standardised by converting all temperature values to the units of Kelvin. This improves the readability of the records and aids in any further analysis. The normalised values and units are added to the database entries to complete the record. If the extracted record has no units, then no conversion is performed at this stage. Any records with incomplete or missing fields are automatically identified and removed so that they do not appear in the final database. Furthermore, this stage merges records that have matching names or labels to create a single record for each compound. Finally, each data entry is tagged with the article DOI, title, authors and journal from which it was sourced.

### Database generation

The ML and NLP process outlined above was applied to all papers in the corpus to generate a database of 39,822 records, consisting of 11,340 and 28,482 Néel and Curie temperature records, respectively. To date, this is the most comprehensive open-source auto-generated magnetic phase transition dataset. The database files were created using the PyMongo database package for the Python programming language. Table 2 describes the modified Snowball algorithm parameters used to generate the database.

### Code availability

All the source code used in this work is made freely available under the MIT license. The code used to generate the database is available at http://github.com/cjcourt/magdb. The updated version version of ChemDataExtractor, including the modified Snowball pipeline, is available at http://github.com/cjcourt/cdesnowball. A clean build of the ChemDataExtractor toolkit is available at http://chemdataextractor.org/download. Further examples of how to use and adapt the toolkit is given at http://magneticmaterials.org/documentation.html.

### Data Records

Static and dynamic forms of the database can be downloaded in MongoDB, CSV or JSON formats through figshare (Data Citation 1) and www.magneticmaterials.org, respectively. The format of the records is given in Table 3. Each contains several fields providing the phase transition temperature of a particular compound and the original source document from which the record was obtained. The *Names* field gives the extracted identifiers of a particular compound, these could be chemical formulae, labels, IUPAC names or other labelling conventions. As described above, a single compound may have several names or labels within a single document, therefore the *Names* field provides all identified names in list format. The *Type* field provides a string indicating the type of phase transition (i.e. Curie or Néel) as determined by either the phrase parsing stages or the modified Snowball algorithm. The *Extracted value* and *Extracted Units* fields give the phase transition temperature and unit of the phase transition as presented in the original source document. During the post-processing stage, the extracted value is converted to units of Kelvin and this normalised temperature is given in the *Normalised Value* field. If the normalisation process was unsuccessful, due to missing units or uninterpretable formatting, then this field, and the *Normalised Units* field are set to be null. The source document fields provide information on the article title, journal, DOI, and authors of the source article.

The *Confidence* field helps to identify phase transitions that have been extracted probabilistically. If the record was retrieved using the modified Snowball algorithm stage, the record contains the associated confidence score determined using Equation 5. If instead the record was produced using deterministic phrase parsing methods, then a confidence score is not assigned, thus giving a null confidence. This null confidence does not indicate zero confidence in the values; it merely reflects the fact that within the scheme of the extraction pipeline, it is not possible to assign a confidence owing to the deterministic nature of these derived values. As the majority of the records in the database are produced in this way, the majority of the records have null confidence.

### Technical Validation

Evaluation of the dataset was realised by taking a random subset of 200 records from the database and comparing them manually to the articles from which they were extracted. Evaluation is made on the basis of precision, calculated using Equation 6. A record was taken to constitute a true positive if all parts of the database record matched to the record found in the article. Any incorrect field meant the record was taken to be a false positive.

(6)Precision=TPTP+FP

This analysis yields an estimated overall precision of 73%, where records were processed via ChemDataExtractor with or without the modified Snowball pipeline as the data necessitated. Similarly, to validate the performance of the modified Snowball algorithm, 200 records produced using the modified Snowball stage were analysed in the same way; these showed a precision of 82%. It is noteworthy that this high level of precision was obtained using only 500 and 370 Curie and Néel seeds, compared to the 90% precision achieved in the original Snowball system, using 4,000 seeds. The vast majority of records were produced via the ChemDataExtractor phrase and table parsing rules; 1,350 records were produced using the modified Snowball pipeline and 38,472 produced using the ChemDataExtractor phrase and table parsers. As such, when taking a random sample of records to evaluate the precision of the database, it is expected that the precision will tend towards the 66% precision estimated for purely phrase and table based parsing, as mentioned in the Background & Summary section.

It should be noted that augmented system has been designed for high precision and low recall. This is because it is more important that the auto-generated records in a materials database are correct than having full coverage. As such, the recall is deemed far less important as an evaluation metric for the dataset presented in this work. However, for reference, the recall of the system was estimated by comparing the output of the adapted ChemDataExtractor system to a manually generated database, derived from a corpus of 50 inorganic chemistry and physics articles. This yielded a recall of 56%.

**Figure 5:**
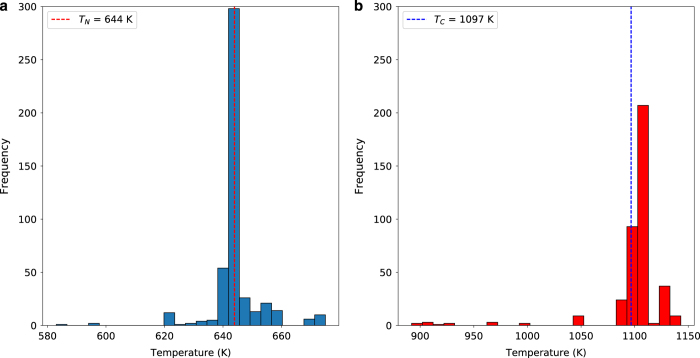
Example Néel and Curie phase transition temperature distributions for *BiFeO*_3_. By analysing all database records containing *BiFeO*_3_, a distribution of extracted phase transition temperatures can be generated and analysed to determine an estimate of the transition temperatures and associated uncertainties. This approach yields average Néel (**a**) and Curie (**b**) temperatures of 644±9*K* and 1,097±32*K*, respectively.

As a case study to highlight the usefulness and validity of the database, Fig. 5 presents the distribution of Curie and Néel phase transition temperatures for the multi-ferroic material *BiFeO*_3_. This material has been widely studied in the physics and inorganic chemistry literature, to the extent that it is the most commonly found inorganic compound in the database. It is present in 920 records. The distribution of Néel temperatures indicates that the reported transition temperatures fall in the range 580–700 K. Similarly the Curie temperature lies in the range 900–1,150 K. Taking a mean and standard deviation of these distributions, and removing values outside one standard deviation, to account for data extraction errors, yields Néel and Curie temperatures of 644±9*K* and 1,097±32*K*, respectively. These values are clearly in-line with the accepted transition temperatures for *BiFeO*_3_ and demonstrate the usefulness of the database for deriving reliable property values and associated uncertainties.

There are still key issues that limit the precision of the database. Firstly, the ChemDataExtractor phrase and table parsing stages fail to distinguish between properties that have overlapping specifiers. For example, the specifier abbreviation *T*_*c*_ is commonly used to identify both a Curie temperature and the superconducting critical temperature. Therefore, many of the Curie temperature records within the database have been incorrectly identified. As magnetism and superconductivity are intrinsically linked, it is the ultimate goal of this research to incorporate all chemical properties associated with magnetism and superconductivity into a single database for the purpose of materials discovery. To do this, future work will look at finding ways to distinguish between properties with overlapping specifiers and classify them appropriately. Secondly, the modified Snowball algorithm stage is only able to perform sentence-level relationship extraction. Therefore, relationships that are spread across paragraphs or whole sections of text cannot be identified with the algorithm presented here. The ChemDataExtractor pipeline attempts to resolve this issue by performing paragraph-level interdependency resolution after all records have been produced (see Fig. 2). However, the complexity of the language and naming conventions used within scientific text makes it very difficult to create a generic interdependency resolution tool that works well on the majority of documents. Future endeavours to overcome this limitation will focus on using probabilistic NLP and ML techniques to identify and track the subject compound entity within the textual elements of scientific documents. Subject identification is a well researched area of NLP. Commonly, methodologies for subject entity identification use noun-verb phrase parsing to create dependency trees from which relationships can be extracted. This approach has led to many successful Open Information Extraction (OpenIE) systems, such as ReVerb^17^ and TextRunner^18^. Such systems are able to identify relationships without any human input. Using a similar approach, the subject compound entity within each sentence of a scientific document could be identified. Then, using a statistical framework for identifying the topic and subject of a paragraph, based on Latent Semantic Analysis (LSA)^19^, it may be possible to probabilistically track the most relevant subject compounds throughout each section. Incomplete records generated by ChemDataExtractor could thereby be assigned to a compound probabilistically in a completely property-independent manner.

### Usage Notes

The database has been provided in multiple formats to make it easier to reuse the data. The JSON and CSV database formats can be easily read using all major programming languages including Python, Java, MatLab and R. The MongoDB files require MongoDB to be installed before use. Instructions on downloading and installing MongoDB can be found at https://docs.mongodb.com/manual/.

The database can be easily queried by compound, phase transition temperature or source article information to form subsets of compounds with desired materials properties. It is therefore also possible to partition the data into sub-classes of materials, such as organic and inorganic compounds. Further normalisation could be performed to standardise the compound names, converting to and from IUPAC codes, smiles, chemical formulae and so on. This would help to combine multiple records for the same compound. As demonstrated in Fig. 5, it is possible to use multiple data entries to derive materials properties. By extension, it is also possible to compare property distributions across classes of properties.

The algorithm and database generation code could be easily extended to include further properties associated with magnetism and superconductivity. Full documentation, demos and examples are available from http://magneticmaterials.org/documentation.html.

## Additional information

**How to cite this article**: Court, C. J. & Cole, J. M. Auto-generated materials database of Curie and Néel temperatures via semi-supervised relationship extraction. *Sci. Data* 5:180111 doi: 10.1038/sdata.2018.111 (2018).

**Publisher’s note**: Springer Nature remains neutral with regard to jurisdictional claims in published maps and institutional affiliations.

## Supplementary Material



## Figures and Tables

**Table 1:** Examples of extraction patterns produced in the training phase of the modified Snowball algorithm.

Relation	Extraction pattern
Curie	of <CEM> ( <Specifier> = <Value> <Unit> )
Curie	<CEM1> and <CEM2> have a <Specifier> of <Value1> and <Value2> <Unit> respectively
Néel	<CEM> is a canted antiferromagnet with <Specifier> = <Value> <Unit>
Néel	temperature <Specifier> = <Value> <Unit> of bulk <CEM> makes

**Table 2:** Input parameters for the modified Snowball algorithm.

Parameter	Description	Value
*τ_sim_*	Minimum phrase object similarity threshold	0.8
*τ_c_*	Minimum relationship confidence threshold	0.8
*v_prefix_*	Prefix element similarity weighting	0.1
*v_middle_*	Middle elements similarity weighting	0.8
*v_suffix_*	Suffix similarity weighting	0.1

**Table 3:** Description of phase transition database records.

Key	Description	Data type
Names	Chemical compound names or labels	List of strings
Type	Transition type	String
Extracted Value	Temperature value in extracted units	String
Extracted Unit	Temperature in Kelvin	String
Normalised Value	Temperature in Kelvin	String
Normalised Unit	Kelvin unit	String
Confidence	Relationship confidence score	Float
Authors	Source document authors	List of Strings
DOI	Source document DOI	String
Journal	Source document journal	String
Title	Source document title	String
